# Sleep, Plasticity and the Pathophysiology of Neurodevelopmental Disorders: The Potential Roles of Protein Synthesis and Other Cellular Processes

**DOI:** 10.3390/brainsci4010150

**Published:** 2014-03-19

**Authors:** Dante Picchioni, R. Michelle Reith, Jeffrey L. Nadel, Carolyn B. Smith

**Affiliations:** 1 Behavioral Biology Branch, Walter Reed Army Institute of Research, Silver Spring, MD 20910, USA; E-Mail: dante.picchioni@nih.gov; 2 Advanced MRI Section, National Institute of Neurological Disorders and Stroke, Bethesda, MD 20892, USA; 3 Section on Neuroadaptation and Protein Metabolism, National Institute of Mental Health, Bethesda, MD 20892, USA; E-Mails: rachel.reith@nih.gov (R.M.R.); jeffrey.nadel@gmail.com (J.L.N.)

**Keywords:** sleep, plasticity, neurodevelopmental disorders, protein synthesis, memory, autism

## Abstract

Sleep is important for neural plasticity, and plasticity underlies sleep-dependent memory consolidation. It is widely appreciated that protein synthesis plays an essential role in neural plasticity. Studies of sleep-dependent memory and sleep-dependent plasticity have begun to examine alterations in these functions in populations with neurological and psychiatric disorders. Such an approach acknowledges that disordered sleep may have functional consequences during wakefulness. Although neurodevelopmental disorders are not considered to be sleep disorders *per se*, recent data has revealed that sleep abnormalities are among the most prevalent and common symptoms and may contribute to the progression of these disorders. The main goal of this review is to highlight the role of disordered sleep in the pathology of neurodevelopmental disorders and to examine some potential mechanisms by which sleep-dependent plasticity may be altered. We will also briefly attempt to extend the same logic to the other end of the developmental spectrum and describe a potential role of disordered sleep in the pathology of neurodegenerative diseases. We conclude by discussing ongoing studies that might provide a more integrative approach to the study of sleep, plasticity, and neurodevelopmental disorders.

## 1. Introduction

Sleep is important for neural plasticity, and plasticity underlies sleep-dependent memory consolidation. Studies of sleep-dependent memory and sleep-dependent plasticity have begun to examine alterations of these functions in populations with neurological and psychiatric disorders. Such an approach acknowledges that disordered sleep may have functional consequences during wakefulness in these disorders. The main goal of this review is to highlight the role of disordered sleep in the pathology of neurodevelopmental disorders and examine some potential mechanisms by which sleep-dependent plasticity may be altered.

We will begin by describing the normal sleep-dependent memory functions that are associated with development. We will then see how sleep deviates from a normal pattern in several neurodevelopmental disorders. Although these disorders are not traditionally considered to be sleep disorders *per se*, recent data has revealed that disordered sleep is among the most consistent symptoms and may contribute to the progression of neurodevelopmental disorders.

We will then outline the molecular mechanisms by which disordered sleep may exert its influence on cognitive symptoms, such as learning and memory impairments. These sections will focus on the molecules and processes that are altered by sleep and by normal and prolonged wakefulness. We will also discuss the effects of these molecules and processes on plasticity. In particular, we will consider protein synthesis, a process essential for plasticity, and the relationship between protein synthesis and sleep.

The final portion of the review will utilize these foundations to describe a possible role of changes in sleep in the pathology of neurodevelopmental disorders. We will also briefly attempt to extend the same logic to the other end of the developmental spectrum and describe a potential role of disordered sleep in the pathology of neurodegenerative diseases. We will conclude by discussing ongoing studies that might provide a more integrative approach to the study of sleep, plasticity and neurodevelopmental disorders.

## 2. Sleep-Dependent Memory during Normal Development

The study of processes that take place during normal sleep is essential to understanding the functions of sleep, and memory consolidation is one such function of sleep. Memory processes are typically categorized into three stages: encoding, consolidation and retrieval. Sleep-dependent memory can be defined as memory consolidation for which encoding took place before sleep and retrieval took place after sleep. Although much remains to be understood and certain manipulations can subtly alter the basic findings [1,2], it is now clear that sleep is essential for the consolidation of certain types of memories. The relevant evidence comes from a large number of studies that were designed to measure sleep-dependent improvements in a variety of memory systems: declarative memory, procedural memory and extinction from classical conditioning. Numerous reviews have been written on the general topic of sleep-dependent memory, but a focus on sleep-dependent memory in infants and children has only emerged within the last year [3]. This is an important research area, because one would expect the importance of sleep for memory consolidation to be particularly high during the periods of intense learning that occur throughout development. On the other hand, one might also expect a complex relationship to exist between sleep-dependent memory and development, because the functions of sleep may vary across the phylogenetic and ontogenetic spectra. This is important to note, because one particular memory process or system may be critical for survival in one species, but essentially unimportant for survival in another species and *vice versa*. Within a species, the same statement may hold for one stage of development *versus* another. For example, a particular skill or behavior may be critical for survival during a certain period of brain development or a certain period of increased risk of predation, but essentially unused when the organism reaches adulthood. Indeed, when examining the literature on sleep-dependent memory during normal development, findings do not often correspond well with the adult literature, and these nuances may be essential to understanding the functions of sleep during development.

Perhaps surprisingly, early in development, sleep appears to hinder memory consolidation [4,5,6]. However, upon closer examination, it becomes clear that the role of sleep in early development may be more complicated. Instead of merely strengthening a memory, the function of sleep early in development may be more closely related to more subtle enhancements of memory, such as memory generalization. As in adults, sleep in children appears to be important for the consolidation of declarative memories (e.g., paired-word associate lists); but in contrast with adults, sleep in children may not be important for the consolidation of procedural or implicit memories (e.g., sequential finger tapping task) [7,8,9,10]. These relationships are complicated by the fact that skill level appears to be important for sleep-dependent memory. Children who have low levels of baseline performance and adults who have high levels of baseline performance on a task do not show a sleep-dependent memory effect; however, children and adults show the same sleep-dependent memory effect for a procedural memory task when equalizing baseline performance to an intermediate level [11]. It seems sleep is less important both for new learners who have never seen the task before and experienced learners who are trying to take their skill to the next level, but it is important for learners at an intermediate skill level regardless of age. In addition, children actually outperform adults when, following sleep, they are asked to extract the explicit components from an implicit learning task [12]. The task that was used to measure both implicit and explicit learning is called the “button-box task”. Subjects were given a box with several buttons with distinct colors. At learning, the buttons illuminated in a fixed sequence, and the subjects were instructed to press each illuminated button as fast as possible. At recall, the procedure was repeated. The time required to press the sequence in its entirety served as the measure of implicit recall. In addition, before repeating the procedure, subjects were asked to state the sequence that they learned in the prior session by slowly pointing at each button in the correct order. The number of correct transitions from one button in the sequence to the next served as the measure of explicit recall.

## 3. Disturbances of Sleep in Neurodevelopmental Disorders

Disorders of brain development are often accompanied by disorders of sleep. The prevalence of abnormal patterns of sleep in neurodevelopmental disorders and the fact that they are associated with more severe behavioral manifestations (selected references [13,14,15,16,17,18]) gives some insight into the importance of sleep for normal brain development.

### 3.1. Autism

Autism spectrum disorder (ASD) is a neurodevelopmental disorder with varying severity. The latest Centers for Disease Control and Prevention report (2008) indicates that the prevalence of ASD is one in 88 children with a 4.6:1 male to female ratio [19]. Diagnosis of ASD is usually made before the age of three and is based on abnormalities in three core components: social interactions, communication and stereotyped repetitive movements [20,21].

Disorders of sleep are one of the most common concurrent clinical disorders in ASD (including pervasive developmental disorder and Asperger’s syndrome), occurring in about 50%–85% of patients [22,23,24,25,26,27,28,29]. The nature of the sleep disturbances varies across patients, but includes decreased total sleep [29,30,31,32,33,34,35], increased sleep latency [26,29,36,37,38,39,40], more fragmented sleep/decreased sleep efficiency [36,38,39,40,41,42], increased stage non-rapid eye movement 1 (N1) sleep [38,40], decreased slow-wave sleep (SWS) [38,40], decreased rapid eye movement (REM) sleep latency [31,35] and decreased REM sleep [33,34,41].

Attaining an accurate estimate of the prevalence of sleep disorders in ASD is difficult, because the patients themselves often do not complain of this problem. Additionally, the caregiver is often more focused on curbing some of the other more debilitating and obvious daytime behaviors. A recent polysomnography study conducted on 17 Asperger’s syndrome or high-functioning ASD patients, excluding subjects with known diagnosis of a sleep disorder, showed that whereas total sleep time did not differ between the ASD group and controls, the subjects with ASD did have significantly increased sleep latency [40]. They also had decreased sleep efficiency and an increase in the percent of time in wakefulness after sleep onset. Sleep stages were also affected, with an increase in the percent of time in N1 sleep and a subsequent decrease in the percent of time in SWS [40]. These results suggest that disordered sleep may persist in many autistic patients whether or not they are aware of it. Therefore, the actual prevalence of sleep abnormalities in patients with ASD may be higher than previously realized.

We can say with relative confidence that many types of memory consolidation take place during sleep. Is there a connection between a lack of sleep and a lack of memory consolidation in pathological conditions that display abnormal sleep patterns as part of their symptom profile? Such pathological conditions include disorders that are traditionally considered sleep disorders [43] and psychiatric disorders, such as depression (e.g., [44]) that are not traditionally considered sleep disorders. Preliminary data on sleep-dependent memory are available for ASD. Sleep-dependent improvements on face perception [45] and associative learning [46] tasks do not appear to be reduced in patients with ASD compared to controls, but correlations between the improvements and sleep quality were only present in controls.

### 3.2. Tuberous Sclerosis Complex

Tuberous sclerosis complex (TSC) affects about 1 in 6000 people [47]. It is caused by an autosomal dominant mutation in either *TSC1* or *TSC2* and is characterized by benign growths throughout the body [47]. Patients with TSC often have seizure disorders (95% of patients), learning disabilities (40%–80%) [48] and ASD (25%–60%) [49,50]. As in ASD, TSC is associated with a high incidence of disordered sleep (30%–90%) [16,51,52,53]. Moreover, a history of seizures is strongly correlated with the severity of sleep disorders in TSC patients [51,52]. Epilepsy is a known risk for sleep abnormalities (selected review [54]). Results of a polysomnography study indicate that patients with TSC and seizures have reduced sleep time, decreased sleep efficiency, increased time awake after sleep onset, decreased REM sleep (both the time and number of REM periods), decreased stage non-rapid eye movement 2 (N2) sleep and increased N1 sleep. A seizure occurring during the polysomnography recording enhanced the effects on the sleep architecture [51].

### 3.3. Fragile X Syndrome

Fragile X syndrome is the most common known cause of cognitive disability and the leading known genetic cause of autism. About 15%–60% of patients with fragile X develop ASD [55,56,57], accounting for about 5% of total patients with ASD [58,59]. Patients with fragile X are usually hyperactive and have problems with attention. Because fragile X is an X-linked disorder, it primarily affects males with a prevalence of about 1 in 4000 [60]. Fragile X syndrome occurs as a result of a trinucleotide (CGG) repeat expansion in the 5′ untranslated region of the fragile X mental retardation (*FMR1*) gene. This expanded repeat sequence leads to silencing of the gene and consequent loss of the protein product, FMRP (fragile X mental retardation protein) [61].

There are several reports of sleep abnormalities in fragile X patients. One parental survey study of 1295 patients reported that 32% of the patients had at least one indication of abnormal sleep, and of these, 84% had two or more abnormalities [17]. However, many fragile X patients (47%) were taking at least one medication to help them sleep [17], suggesting that sleep abnormalities may be more common than indicated by the survey. Based on sleep diaries, patients with fragile X were found to have increased sleep latency (*p* = 0.01), increased time awake after sleep onset (*p* = 0.03) and a trend toward decreased total sleep time (*p* = 0.09) [62]. Small-scale polysomnography studies have shown that fragile X patients have reduced total sleep [63], increased REM latency [63], reduced REM sleep [63,64] and increased N1 [64] and SWS [63].

Studies in a *Drosophila* model of fragile X (*dfmr1*) reveal that mutants sleep more than wild-type controls (mainly during the active phase), whereas hypermorphs sleep less than wild-type controls [65]. This finding contrasts with the deficiencies in sleep in the human disease, suggesting a complex role of FMRP in sleep. We note, however, that *Drosophila* lack the mammalian homologs of FMRP, FXR1P and FXR2P. Mice that lack both FMRP and FXR2P have abnormal circadian rhythm, suggesting that the combination of these proteins may play a role in sleep behavior [66]. In a recent study of cortical activity in developing *Fmr1* knockout mice [67], higher rates of synchrony and increased firing were seen during sleep compared to controls. Because these differences in neuronal firing were seen during sleep and during a critical period of plasticity, the authors suggest that even if *Fmr1* knockout mice have adequate neuronal function during wakefulness, the hyperexcitability of neuronal networks during sleep may have an adverse effect on plasticity [67].

### 3.4. Rett Syndrome

Rett syndrome is caused by mutations in the coding region of the methyl CpG binding protein 2 (*MECP2*) gene. MeCP2 is involved in gene silencing [68]. Rett syndrome occurs almost exclusively in female patients with a prevalence of 1 in 15,000 female births [68]. Rett syndrome leads to cognitive impairments, seizures and autistic-like behaviors, such as a lack of communication, impaired social interaction and repetitive stereotyped hand movements [68].

In a study conducted by Marcus and colleagues, nighttime polysomnography did not differ between 30 Rett syndrome patients and 30 controls [69]. Carotenuto and colleagues compared 13 patients with Rett syndrome and 40 controls and found that Rett patients had decreased sleep efficiency, increased SWS and decreased REM sleep (though sleep latency and total sleep time were unaffected) [70]. In a population of 20 girls with Rett syndrome, Piazza and colleagues showed that they had decreased sleep at night and a subsequent increase in sleep during the day [71]. In an actigraphy study, McArthur and colleagues showed that nine patients with Rett syndrome had decreased total sleep time, increased sleep latency and more fragmented/less efficient sleep compared to controls [72]. While these studies do not necessarily concur on the nature of the sleep abnormalities in Rett syndrome patients, they indicate that sleep is affected in this disease.

Transgenic mouse models of Rett syndrome (Mecp2^−/−^) recapitulate many of the features observed in the human disorder, including abnormal social interactions [73], increased anxiety [74] and abnormal hind limb clasping (analogous to hand flapping in patients) [75]. Moreover, home-cage monitoring of Mecp2^−/−^ mice showed decreased activity during the active cycle [73,74] and increased activity during the rest cycle [73], suggesting that Rett syndrome mice sleep less during the rest cycle and more during the active cycle. This phenotype is reminiscent of the findings in patients [71,72].

### 3.5. Prader–Willi Syndrome

Prader–Willi syndrome is a neurodevelopmental disorder with a prevalence of about 1 in 10,000–25,000. It occurs as a result of the loss of expression of a segment of genes on the paternal chromosome, 15q11-13. Features of this disorder include short stature, low muscle tone, cognitive impairments, behavioral abnormalities and excessive hunger, leading to overeating and obesity [76,77]. Other behavioral phenotypes include language abnormalities [78], repetitive behaviors similar to obsessive compulsive disorder [79] and social behavior abnormalities [79]; characteristics also found in ASD. Many patients with Prader–Willi syndrome have disordered sleep [80]. A polysomnography study showed that patients with Prader–Willi syndrome have decreased sleep latency, decreased REM latency and decreased SWS [81]. Decreased sleep latency has been confirmed in an actigraphy study [82]. Most commonly, patients with Prader–Willi syndrome are reported to have excessive daytime sleepiness compared to neurotypical controls [81,82,83,84,85,86,87]. In a study of 21 patients with Prader–Willi syndrome, 95% had excessive daytime sleepiness [83] and 52% had sleep-onset REM periods [83], which have also been reported in other studies [84,88,89]. In addition, patients with Prader–Willi syndrome have been reported to have decreased levels of hypocretin in cerebrospinal fluid (CSF) [85]. Whereas excessive daytime sleepiness, sleep-onset REM and decreased levels of hypocretin in CSF are also found in narcolepsy, cataplexy and hypnogogic hallucinations (features also associated with narcolepsy) are not commonly observed in Prader–Willi patients [89,90]. Other studies suggest that the apnea-hypopnea index is higher in patients with Prader–Willi syndrome and correlates with body mass index [81].

### 3.6. Angelman Syndrome

Angelman syndrome (prevalence of about 1 in 10,000) [91,92] is historically labeled as the “happy puppet” syndrome, because of the patients’ typically happy demeanor. Patients with Angelman syndrome often exhibit severe intellectual disability, seizures, speech impairment and stereotypical behaviors. They also often meet the criteria for ASD [91,93]. Angelman syndrome is a disorder of imprinting that is caused by silencing of the maternally inherited region on chromosome 15q. Disturbances of sleep are a common feature of Angelman syndrome, affecting 20%–90% of patients, and are particularly evident in young children [80,94,95,96,97,98,99]. Compared with control subjects, patients with Angelman syndrome have reduced total sleep time [96,99], increased sleep latency [96,99,100], poor sleep quality, including increased wakefulness after sleep onset [99,100], reduced REM sleep [99,100], increased SWS [99] and excessive daytime sleepiness [96]. They also often have hyperkinesias, including increased periodic limb movements [99] and an increased incidence of sleep-walking [96].

Even compared to patients with intellectual disability and epilepsy, patients with Angelman syndrome still had increased sleep latency, increased wakefulness after sleep onset and a trend toward increased periodic leg movements [100], suggesting that sleep abnormalities in Angelman syndrome extend beyond the effects of intellectual disability and epilepsy.

The gene ubiquitin ligase E3A (UBE3A) is one of the critical genes in the chromosomal 15 region associated with Angelman syndrome. Rarely, Angelman syndrome can occur as a result of an isolated mutation on UBE3A; these patients also have sleep disturbances [101]. In Ube3a knockout mice, many features associated with Angelman syndrome are recapitulated [102], including disturbances of sleep, such as decreased time in REM sleep [103].

### 3.7. Williams Syndrome

Williams syndrome is a neurodevelopmental disorder, with a prevalence of about 1 in 7500, caused by a deletion in chromosome 7q11.23. It is characterized by dysmorphic “elfin” facial features, developmental delay and behavioral abnormalities [104]. Almost the antithesis of autism, patients with Williams syndrome are characterized as overly social and have highly expressive language relative to their cognitive development [105,106,107].

There have been a few reports indicating that patients with Williams syndrome have disorders of sleep (reviewed by [80,108]). Questionnaire-based studies indicate that patients with Williams syndrome have bedtime resistance [109], difficulty falling asleep [110], increased sleep latency [109,111], decreased sleep efficiency/increased night awakenings [109,110,111], increased periodic leg movements [110,111] and excessive daytime sleepiness [109]. More objective measures of sleep (actigraphy and polysomnography) indicate that patients with Williams syndrome have reduced total sleep time [112] (however, other studies have shown that total sleep time was not different from controls [113,114]), increased sleep latency [113] (however, another study showed that this did not differ from controls [114]), decreased sleep efficiency/increased wakefulness after sleep onset [110,112], abnormal electroencephalography (EEG) spectral power distributions [115], decreased REM sleep [112], decreased N1/N2 sleep [114], increased SWS [110,114] and increased periodic leg movements [112].

### 3.8. Down Syndrome

Down syndrome occurs as a result of a third copy of chromosome 21 causing characteristic facial features, intellectual disability and numerous other complications, including risk for cardiovascular disease and Alzheimer’s disease [116]. Sleep abnormalities are observed in numerous cases of Down syndrome [113,117,118,119,120,121,122]. The reported sleep abnormalities are: decreased total sleep [123], increased wakefulness after sleep onset/decreased sleep efficiency [64,113,121,122], increased N1 sleep [64], decreased N2 sleep [64,120,122], increased S4 sleep [120], increased latency to REM [120], decreased REM sleep [64] and excessive daytime sleepiness [123,124,125]. In addition, many studies have reported increased prevalence of sleep apnea (selected references [119,126,127]). Decreased total sleep (particularly, REM sleep) has also been shown in a mouse model of Down syndrome (Ts65Dn) [128]. The sleep abnormalities and impairments in long-term memory in this model are both reversed in a transgenic variation (Ts65/App^++−^) in which a critical component of the mutation (App) is present at a normal dosage [129].

### 3.9. Attention-Deficit/Hyperactivity Disorder

In addition to deficits in attention, patients with attention-deficit/hyperactivity disorder (ADHD) also exhibit deficits in memory [130] and prominent sleep abnormalities [131]. In a study of medication-free ADHD patients and healthy controls (9–16 years of age), sleep-dependent consolidation of declarative memories was absent in the ADHD patients [130], but sleep-dependent consolidation of procedural memories, which is not seen in healthy children, was present in patients with ADHD [130]. This finding may be a function of the known prefrontal cortex alterations in ADHD and a competition between implicit and explicit memory consolidation during sleep [3]. The prefrontal cortex plays a prominent role in theories of explicit memory consolidation (e.g., [132]). Wilhelm and colleagues proposed that if sleep can only consolidate one memory system at a time, and ADHD patients experience impairment in sleep-dependent explicit memory consolidation due to alteration in the prefrontal cortex, this impairment may paradoxically improve their sleep-dependent implicit memories. These initial studies are consistent with the importance of sleep-dependent memory in neurodevelopmental disorders and may point to a role of sleep disorders in the accompanying cognitive disabilities. We will next discuss potential mechanisms underlying sleep-dependent alterations in memory to see how they intersect with neurodevelopmental disorders.

## 4. The Cellular Consequences of Normal and Prolonged Wakefulness and Their Effects on Plasticity

Sleep is hypothesized to play a key role in plasticity. Sleep deprivation is associated with deleterious effects on long-term potentiation (LTP), and increases in synaptic strength during sleep have been observed in several studies (reviewed by [133,134,135,136]). However, the exact cellular mechanisms by which this may occur are still being investigated. We will discuss the cellular consequences of normal and prolonged wakefulness that may underlie alterations in plasticity in this section.

### 4.1. Sleep Regulatory Substances

The drive for sleep at any point in time is determined by two processes: a circadian process that varies in a sinusoidal manner and a homeostatic process that increases in a logarithmic manner during sustained wakefulness [137,138]. The homeostatic process is mostly confined to the description of non-REM sleep, whereas REM sleep is mostly controlled by circadian processes (e.g., [139]). In the homeostatic control of non-REM sleep, several substances have been proposed to underlie the signal in the brain, and these substances have been labeled as homeostatic sleep factors or sleep regulatory substances [140]. Among the criteria that are used to classify something as a sleep regulatory substance are: (1) it should accumulate endogenously during periods of sustained wakefulness; (2) it should dissipate endogenously during subsequent sleep; and (3) when administered exogenously, it should induce sleep. These sleep regulatory substances are paramount when considering the cellular consequences of sleep deprivation and their effects on plasticity, because they are inherently linked to sleep regulation.

#### 4.1.1. Adenosine

The classic sleep regulatory substance is adenosine. The theory behind adenosine as a sleep regulatory substance begins with the importance of adenosine triphosphate in the cellular energy cycle. In addition to reentering the energy cycle, adenosine diphosphate is also further metabolized into adenosine monophosphate and, eventually, adenosine. It can then be transported into the extracellular space, where it can act as a neurotransmitter. During wakefulness, when neural energy demands are high, adenosine builds up in the extracellular space, and this buildup is thought to be a neurochemical signal for the homeostatic sleep drive.

The evidence for adenosine as a sleep regulatory substance is substantial [141]. Microdialysis studies in freely behaving cats and rats show that adenosine increases in basal forebrain and cortex during normal wakefulness and wakefulness during sleep deprivation [142,143,144] and that adenosine decreases in the basal forebrain during subsequent recovery sleep. Moreover, adenosine administered by microdialysis into the basal forebrain increases sleep in cats and rats [142,145]. Adenosine is thought to act through inhibitory receptors in the basal forebrain, and this is consistent with the wake-promoting role of this region [146]. A sleep-promoting action of adenosine administered at other sites in the brain is not well established. Adenosine concentration also increases in other parts of the brain during wakefulness. For example, adenosine has been shown to increase in the hippocampus [147]. The hippocampus is rich in A1 receptors [148], and the activation of A1 receptors is known to dampen LTP [149]. This raises the possibility of adenosine as mediating a molecular link between sleep and plasticity [150].

#### 4.1.2. Prostaglandins

Prostaglandin D2 (PGD2) plays a role in endogenous sleep promotion, inducing both REM and non-REM sleep (selected review [151]), possibly mediated by adenosine [152]. In an *in vitro* model, PGD2 dramatically increased secretion of nerve growth factor (NGF) and brain-derived neurotrophic factor (BDNF), which are important for neuronal growth and survival [153]. These neurotrophins are also thought to promote myelination (selected review [154]) (see section 6.2.2 for a review of sleep regulation by myelination), which may be another mechanism by which they affect plasticity. The neurotrophin-mediated effects of sleep-promoting PGD2 support the idea that sleep may fulfill a restorative role in the maintenance of brain structure.

#### 4.1.3. Nitric Oxide

It is widely accepted that nitric oxide facilitates sleep (selected reviews [155,156]). Inhibition of nitric oxide synthase selectively inhibits REM sleep by decreasing acetylcholine release in the pons [157]. Nitric oxide acts as a vasodilator [158]. Increased blood flow in the brain leads to increased oxygen availability and may improve plasticity in certain cases. Moderate levels of nitric oxide can protect cells against damage from oxidative stress [159,160], thereby reducing the associated neurotoxicity and potentially affecting plasticity. Nitric oxide is also thought to act as a second messenger in the cell facilitating LTP when paired with weak electrical stimulation [161]. LTP is inhibited with nitric oxide synthase inhibition [162].

#### 4.1.4. Cytokines

Cytokines, such as Interleukin 1 (IL-1) and tumor necrosis factor-alpha (TNFα), have sleep-promoting effects (selected reviews [140,163]). These two cytokines also stimulate the production of each other [164,165], and IL-1 is hypothesized to increase the synthesis of adenosine [166], PGD2 [167,168] and nitric oxide [169]. Interestingly, both of these cytokines appear to have effects on plasticity [170,171]. IL-1 expression increases during LTP, and inhibition of IL-1 inhibits LTP, showing that IL-1 expression is necessary for LTP (both *in vitro* and *in vivo*) [170]. Similarly, reduced IL-1 signaling leads to deficits in hippocampal-dependent learning and memory [172]. TNFα plays a critical role in the homeostatic regulation of cortical plasticity [171] and promotes synaptic upscaling [173]. The mechanisms underlying the effects of IL-1 and TNFα may be similar because of the bidirectional interactions between these two cytokines.

### 4.2. Sleep Regulatory Processes

**Figure 1:**
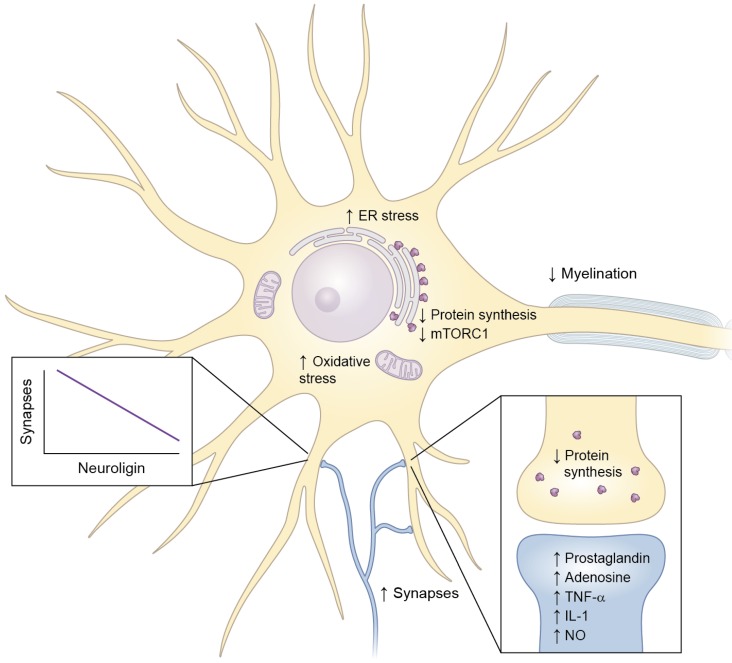
Hypothetical scheme illustrating cellular consequences of sleep deprivation. Prolonged wakefulness leads to the activation of cellular stress events: Endoplasmic reticulum (ER) stress and oxidative stress are activated. Myelination is downregulated. Protein synthesis (in addition to mTORC1 activity) is diminished (both globally and at synapses). Despite these changes, net synaptic upscaling occurs without proper pruning. There is also a possible relationship between the increased number of synapses and decreased neuroligin following sleep deprivation. In addition, accumulation of sleep regulatory substances in the post-synaptic terminal may lead to an increased drive for sleep.

In addition to the classical sleep regulatory substances, there are also cellular processes that are affected by sleep that may serve to homeostatically regulate sleep. These are summarized in Figure 1.

#### 4.2.1. Synapse Formation and Synaptic Activity

The synaptic homeostasis hypothesis proposes that sleep is needed for synaptic pruning. It asserts that wakefulness globally promotes synapse formation, and that sleep is associated with the downregulation/pruning of these connections allowing for strengthening and energy conservation [174,175]. Protein expression studies indicate that sleep is associated with decreased expression of synaptic markers [176,177], and imaging studies indicate that there is a net elimination of dendritic spines during sleep [178,179,180,181]. Conversely, wakefulness is associated with a net increase in synapse size and number [178,179,180,181]. There are limitations to the synaptic homeostasis hypothesis [182]. In particular, synaptic plasticity induced by monocular occlusion in the developing binocular visual system is accompanied by sleep-dependent enhancement in the neuronal firing rate and synaptic strengthening [183].

Neuroligins are cell adhesion proteins that facilitate synaptogenesis, regulating excitatory/inhibitory connections. The neuroligin family is comprised of five related genes (four in mice), *NLGN1*–*NLGN5* [184,185,186,187,188]. They are recruited to the developing synapse and are thought to be more involved in synapse maturation rather than synapse formation. A knockout mouse model of neuroligin1-3 (triple knockout) had deficits in synaptic maturation, but surprisingly had a comparable number of synaptic puncta compared to control mice [189].

The neuroligin gene family has been implicated in the development of autism [190,191,192]. Some studies show that the expression of neuroligin 1 and 2 is decreased following sleep deprivation [54,193,194]. The diminishing availability of neuroligin may be an epiphenomenon or it may actually drive the need for sleep in the animal. Indeed, neuroligin-1 mutant mice have increased non-REM sleep and are difficult to keep awake during a sleep deprivation paradigm [195]. Furthermore, neuroligin-4 mutant *Drosophila* have decreased sleep compared to controls [196]. It would be interesting to know if the overexpression of neuroligin affects wakefulness. The connection between neuroligin and plasticity is well established. Neuroligin-1 is required for normal LTP [197]; deletion of neuroligin-1 results in impaired spatial memory [198], and overexpression of neuroligin-1 induces learning deficits [199].

#### 4.2.2. Myelination

Myelin, produced by oligodendroglia, is critical for the proper development of the nervous system, because it affects the speed at which electrical impulses are propagated. Gene expression studies have shown that myelin-related genes are induced during sleep [200,201,202] and are downregulated after sleep deprivation [203,204]. Myelin basic protein (MBP), a major constituent of myelin, is expressed in large quantities prior to myelination, and the levels of MBP decrease during myelination as the protein is incorporated into the myelin sheath [205]. Injection of MBP into the cat brain reduced the latency to REM sleep and increased the number of REM periods [206]. A recent study showed that sleep (particularly REM sleep) is important for the proliferation of immature oligodendrocytes and that differentiation of oligodendrocytes primarily occurs during wakefulness [202]. Upon firing of an action potential, the sleep-promoting factor, adenosine (Section 4.1.1), activates signaling involved in promoting the differentiation/maturation of mature oligodendrocytes and the formation of the myelin sheath [207].

In narcolepsy, a profound sleep disorder, abnormalities in white matter are reported [208]. A missense mutation in myelin oligodendrocyte glycoprotein was determined to be the cause of a familial form of narcolepsy [209]. This mutation is also linked to multiple sclerosis [209], a disorder of progressive demyelination, which is associated with sleep disorders, such as hypersomnia (which is closely related to narcolepsy), insomnia, sleep-related movement disorders and parasomnias (reviewed in [210]). Poor sleep quality exacerbates the symptoms associated with multiple sclerosis, and symptoms improve after a nap [211]. In a demyelinating rat model (*taiep* rats), REM sleep is abnormal [212], and *taiep* rats have periods of immobility with sleep-onset REM periods similar to narcolepsy [213].

Myelination is believed to play a significant role in cortical plasticity [214,215]. Visual system deprivation results in decreased myelination [216], whereas premature eye opening results in accelerated myelin formation [217]. Social enrichment is another known inducer of cortical plasticity [218]. Rats exposed to social enrichment demonstrate sex-specific effects on myelination. Socially-enriched males have an increased diameter of myelinated fibers, whereas socially-enriched females have an increased number of myelinated axons [219].

Not only is myelination an important accompaniment of cortical plasticity during early development, but myelination continues through adolescence and into adulthood [220,221,222,223,224,225,226]. Studies also suggest that the corpus callosum (one common primary site for oligodendrocytes) continues to grow through adulthood (mid-20s in humans) [227]. The role of myelination on cortical plasticity in adulthood is not as well examined, but it has been suggested that late-life learning can induce myelin formation in adults. Extensive piano practicing was correlated with increased myelination in all age ranges (childhood, adolescence and adulthood) [228].

#### 4.2.3. Cellular Toxicity

##### 4.2.3.1. The Glymphatic System

An emerging hypothesis of the restorative function of sleep is that sleep enhances the clearance of degradative products of neuronal activity through the glymphatic system. The glymphatic system is a unique pathway by which the brain clears waste products and metabolites [229]. Both the volume of interstitial space in brain and the exchange of interstitial and cerebrospinal fluid are considerably increased during sleep [230]. It is hypothesized that neuronal activity triggers the release of toxic degradation products, which are only cleared by this increased fluid exchange when the brain is in a resting state during sleep [230]. This hypothesis offers an explanation for why prolonged periods of wakefulness may lead to cellular damage and ultimately can lead to death of the organism. Though the glymphatic system is newly described, the role of sleep has long been thought to facilitate neuronal detoxification [231]. There is also compelling evidence that sleep has a restorative function in other cellular stress-mediated pathways, as discussed below.

##### 4.2.3.2. Unfolded Protein Response (UPR)/Endoplasmic Reticulum (ER) Stress

The endoplasmic reticulum (ER) is an important cellular site for the synthesis, folding and processing of proteins. Accumulation of unfolded or misfolded proteins (either through normal cellular function or a disease-associated process) in the ER triggers an adaptive response called the unfolded protein response (UPR). This acts to initiate three steps: (1) pause translation; (2) increase production of chaperone proteins to increase protein folding; and (3) promoting the degradation of the excess misfolded proteins [232]. If prolonged, UPR can lead to ER stress and initiate a pro-apoptotic cascade through induction of CHOP (CCAAT/enhancer-binding protein homologous protein) [233]. ER stress can also lead to changes in the calcium messenger system and, thus, alter neuronal plasticity [234].

In normal animals (*Drosophila*, mice, rats and sparrows), short-term sleep deprivation of five to eight hours leads to significant upregulation of the components of the UPR, including receptors (PERK (protein kinase RNA-like endoplasmic reticulum kinase)) (mRNA) [235], translational regulators (*p*-eIF2α (eukaryotic initiation factor 2α)) (mRNA) [200,235,236], chaperone proteins (BiP (binding immunoglobulin protein), PDI (protein disulfide isomerase) or heat shock proteins) (mRNA and protein) [202,204,235,237,238,239,240,241,242,243,244,245] and pro-apoptotic inducers (CHOP) (mRNA) [246]. It should be noted that two studies did not find upregulation of UPR after sleep deprivation [247,248].

If BiP is overexpressed after sleep deprivation, it leads to an increase in subsequent recovery sleep. Conversely, impairment of functional BiP leads to decreased recovery sleep in *Drosophila* [245]. In addition, modulation of UPR by inhibiting the dephosphorylation of eIF2α (thereby inhibiting translation) through treatment with Salubrinal, increased non-REM sleep [249]. When administered during recovery sleep, Salubrinal increased the time spent in recovery sleep [250]. It seems that not only does abnormal sleep affect UPR, but the UPR pathway may also drive the need for sleep.

##### 4.2.3.3. Oxidative Stress

The production of free radicals is a normal part of metabolism, and there are cellular mechanisms to remove these reactive oxygen species (ROS). If the ROS production rate is higher than the capability of the cell to clear the free radicals, this burden results in oxidative stress [251]. The UPR/ER stress pathways are tightly linked with oxidative stress. Hypoxic conditions generating oxidative stress can induce UPR/ER stress [252,253], and antioxidants decrease ER stress [254]. In addition, UPR/ER stress can modulate oxidative stress [255,256].

Many studies have indicated that the oxidative stress pathway is induced with acute, total sleep deprivation (both total and REM sleep) and chronic, partial sleep deprivation [200,257,258,259,260,261,262,263,264,265] and that recovery sleep leads to the restoration of cellular antioxidants [263]. However, these effects are not universally replicated [266,267].

The effect of ROS on LTP is complex; ROS may both facilitate normal LTP and inhibit it in aged animals [268]. Interestingly, melatonin can act as an antioxidant by scavenging and absorbing free radicals [269,270,271,272]. It is not known whether the antioxidant effects and the sleep-inducing effects of melatonin are linked. Endogenous melatonin levels are sensitive to light exposure [273], and disrupted sleep patterns may alter the expression of melatonin, possibly leading to an increase in ROS [271]. The results of studies in mice and rats suggest that sleep deprivation-induced memory impairments may occur through the induction of oxidative stress [261,265,274].

##### 4.2.3.4. Neurodegeneration Following Cellular Stress

Why does sleep deprivation lead to these cellular stress events, and what are the downstream consequences of the activation of stress pathways? The initial induction of the UPR is not necessarily deleterious and might actually protect from the negative effects of sleep deprivation. Studies in *Drosophila* indicate that sleep deprivation induces chaperone proteins that may be protective [275]. Ten hours of sleep deprivation is lethal in the *Drosophila* mutant, *cyc*, in which the induction of chaperone proteins is dampened. Activation of these chaperone proteins before sleep deprivation rescues the lethality associated with sleep loss [275]. Moreover, a *Drosophila* mutant lacking a functional heat shock protein (Hsp83) has a similar phenotype [275]. Sleep deprivation-induction of chaperone proteins may be an attempt at the cellular level to protect from the toxic effects of sleep deprivation [275,276]. If these altered states are maintained for long enough periods or combined with the reduced ability of the cell to adapt, increases in ER and oxidative stress can ensue. These cell stresses can ultimately progress to apoptosis. Whether neurodegeneration occurs following sleep deprivation remains controversial, with some studies reporting that sleep deprivation (both total and REM sleep) leads to the upregulation of apoptotic genes and increased cell death [194,202,203,204,277,278,279] and others reporting no evidence for degeneration by either morphological or expression data [248,280].

## 5. The Role of Protein Synthesis in Sleep-Dependent Plasticity

### 5.1. Transcription *versus* Translation

Sleep is associated with increased cortical expression of numerous transcripts that indicate differences between the functional categories of genes expressed during sleep and wakefulness (e.g., [200,240]). In rat cortex, sleep was associated with increased mRNA levels of key translational components, such as eukaryotic translation elongation factor 2 (eEF2) and initiation factor 4AII (eIF4αII). Other genes with increased expression during sleep included genes involved in membrane trafficking, membrane maintenance/synthesis, cholesterol synthesis, consolidation of memory, such as calmodulin-dependent protein kinase IV, and some aspects of plasticity, such as calcineurin. Although these effects on gene expression are suggestive that sleep may actively support specific cellular functions, changes in the levels of transcripts do not map directly to functional changes. Functional changes are only determined by changes in translational and post-translational events, and sleep has been proposed to play a prominent role in these events [281,282]. Therefore, this section will focus on sleep studies designed to manipulate or measure protein synthesis directly. We will also limit our discussion of sleep studies designed to measure protein levels or post-translational events to those with a direct impact on protein synthesis. Readers interested in the importance of the expression of specific proteins in sleep-dependent plasticity are directed to other excellent reviews on this topic (e.g., [283]).

### 5.2. Manipulating Protein Synthesis

The data on the effects of administering protein synthesis inhibitors on sleep are somewhat contradictory. For a summary of this literature, see Table 1, which is organized according to the inhibitor used in each study. Most of these studies were performed in rats and cats, and some were performed in mice and humans. One specific issue to keep in mind is the relative extent to which each drug crosses the blood-brain barrier. Although this is not relevant to the studies that administered the drug directly into the brain, further scrutiny of blood-brain barrier permeability may explain some of the discrepant findings.

In general, about half of the studies in which inhibitors were administered systemically reported decreased REM sleep. Site-specific administration of anisomycin into the ventrolateral preoptic hypothalamic nucleus or the hippocampus had no effect on sleep, whereas injection into the lateral hypothalamus resulted in increased REM sleep. Following intracerebroventricular administration of inhibitors, half of the studies reported increased non-REM sleep. In particular, intracerebroventricular administration of Salubrinal, an eIF2α phosphatase inhibitor, was effective in increasing non-REM sleep. Salubrinal, which also enhances the UPR, may result in increased non-REM sleep through an increase in ER stress. These results, among other results in the literature, were used to propose the following theory on the role of ER stress in sleep homeostasis. During wakefulness, there is an increase in ER stress. This signals the need for sleep and the associated synthesis of new proteins involved in protein folding. Therefore, if you inhibit protein synthesis with Salubrinal, you potentiate the need for sleep by increasing ER stress.

**Table 1:** Summary of the literature on the effect of protein synthesis inhibitors on sleep.

Year, First Author	Inhibitor	Administration Method/Location	NR	R
1975, Drucker-Colin [284]	Anisomycin	Intraperitoneal	0	–
1977, Rojas-Ramirez [285]	Anisomycin	Intraperitoneal	0	–
1979, Drucker-Colin [286]	Anisomycin	Intraperitoneal	0	–
1980, Gutwein [287]	Anisomycin	Subcutaneous	+	–
2008, Methippara [288]	Anisomycin	Ventrolateral Preoptic Nucleus	+	0
2008, Methippara [288]	Anisomycin	Lateral Hypothalamus	0	+
2008, Methippara [288]	Anisomycin	Hippocampus	0	0
1975, Kitahama [289]	Chloramphenicol	Oral	–	–
1977, Rojas-Ramirez [285]	Chloramphenicol	Intraperitoneal	0	–
1979, Drucker-Colin [286]	Chloramphenicol	Intraperitoneal	+	–
1979, Petitjean [290]	Chloramphenicol	Oral	–	–
1980, Drucker-Colin [291]	Chloramphenicol	Oral	0	–
1982, Bowersox [292]	Chloramphenicol	Oral	0	–
2005, Moulin-Sallanon [293]	Chloramphenicol	Intraperitoneal	–	–
1972, Stern [294]	Cycloheximide	Intracerebroventricular	+	+
1972, Stern [294]	Cycloheximide	Intraperitoneal	–	0
1973, Pegram [295]	Cycloheximide	Subcutaneous	+	–
1981, Uezu [296]	Cycloheximide	Intracerebroventricular	0	–
1981, Uezu [296]	Cycloheximide	Intraperitoneal	0	–
1979, Petitjean [290]	Erythromycin	Oral	0	–
1983, Nonaka [297]	Minocycline	Oral	–	0
1979, Petitjean [290]	Oxytetracycline	Oral	0	0
1977, Rojas-Ramirez [285]	Penicillin G	Intraperitoneal	0	0
1972, Stern [294]	Puromycin	Intracerebroventricular	0	0
1972, Stern [294]	Puromycin	Intraperitoneal	0	0
1981, Uezu [296]	Puromycin	Intracerebroventricular	0	–
1981, Uezu [296]	Puromycin	Intraperitoneal	0	0
2009, Methippara [249]	Salubrinal	Intracerebroventricular	+	0
2012, Methippara [250]	Salubrinal	Intracerebroventricular	+	0
1975, Kitahama [289]	Thiamphenicol	Oral	0	0
1979, Petitjean [290]	Thiamphenicol	Oral	0	0
1982, Bowersox [292]	Thiamphenicol	Oral	0	0

Notes: (NR) = the effect on non-REM sleep; (R) = the effect on REM sleep; (+) = increase; (–) = decrease; (0) = no effect.

In contrast to the large number of substances that decrease protein synthesis, fewer substances, such as growth hormone and leucine, are known to increase protein synthesis in some tissues. Whether they have similar effects in brain has not been demonstrated. The release of growth hormone occurs during non-REM sleep [298]. Intraperitoneal administration of growth hormone has no effect on non-REM sleep, but significantly increases REM sleep [284]. Growth hormone-releasing hormone increases non-REM sleep in a variety of species and with a variety of administration methods [140]. In a study in healthy human subjects, an infusion of branched chain amino acids, including leucine, had no effects on any of the sleep parameters analyzed [299]. For example, the percentages of SWS during the nights of administration of branched chain amino acids and placebo were 14% (*SD* = 6%) and 13% (*SD* = 8%), respectively, and the percentages of REM sleep during the nights of administration of branched chain amino acids and placebo were 20% (*SD* = 5%) and 19% (*SD* = 4%), respectively.

### 5.3. Measuring Protein Synthesis: Sleep Deprivation

Instead of manipulating protein synthesis and measuring the subsequent effects on sleep, other studies were designed to manipulate sleep and measure the effects on protein synthesis. The manipulation of sleep can take the form of a sleep-deprived *versus* rested comparison, a wakefulness *versus* sleep comparison or some combination thereof. An example of the last scenario is a comparison of protein synthesis during rested wakefulness, sleep-deprived wakefulness and sleep. We will begin by reviewing studies that included some type of sleep deprivation.

Using the incorporation of tritiated amino acids as a measure of protein synthesis, Bobillier and colleagues [300] performed a series of experiments that involved sleep deprivation. In seven-day-old rats, they compared 1.5 hours of total sleep deprivation by gentle handling to control animals that were given the opportunity to spontaneously fluctuate between sleep and wakefulness. Sleep-deprived animals were studied during wakefulness, but it is not possible to know the state in which the control animals were studied, due to the nature of the control condition. No differences were observed in protein synthesis. In adult rats, they compared 48 hours of selective REM sleep deprivation to control animals. REM deprivation was accomplished with the “inverted-flower-pot” method. In this method, investigators placed the animals on a small platform surrounded by water. Whenever the animal entered REM sleep, which was accompanied by the loss of skeletal muscle tone, it fell into the water. Using this manipulation, the investigators observed a decrease in protein synthesis, an observation that replicated a similar previous study [301]. This finding was not replicated when control animals were exposed to all experimental conditions except the actual REM deprivation [302]. This was accomplished by placing the animals on a slightly larger platform so that low skeletal muscle tone did not cause them to fall into the water. Therefore, the effects of sleep-deprivation on brain protein synthesis remain an open question.

### 5.4. Measuring Protein Synthesis: Normal Sleep

The study of the effects of sleep deprivation has a long history and has clear practical implications. In addition to removing sleep and observing the subsequent deleterious consequences, it is also important to study the processes that accompany normal sleep. Early studies, in which incorporation of [3H]leucine into protein was assessed, reported higher levels of incorporation during REM sleep compared to non-REM sleep [303,304], but no difference between wakefulness and non-REM sleep [305]. It is important to note, however, that the incorporation of exogenously-administered radiolabeled amino acids into protein may or may not reflect the actual rates of tissue protein synthesis. Incorporation of labeled amino acids into protein may be influenced by the rates of clearance of the labeled amino acids, the endogenous levels of unlabeled amino acids, the rates of the recycling of unlabeled amino acids, and more. The advent of the quantitative autoradiographic l-[1-^14^C]leucine method made it possible to avoid these potential sources of error and measure incorporation rates of unlabeled leucine into tissue protein [306]. Subsequent studies have used this method in conjunction with quantitative autoradiography to examine the effects of sleep state on regional protein synthesis rates in the brain [307,308,309].

In a study of REM-deprived adult rats, Ramm and Smith [309] found that weighted time in non-REM, but not REM, sleep was positively correlated with the rate of protein synthesis in most regions of the brain. Similar results were reported in a study of sleep-sated, adult rhesus monkeys [308]. For deep sleep, correlations in all 57 regions examined were positive, and most were either statistically significant or close to significant. The investigators concluded that the specificity of the correlations to deep sleep suggests that protein synthesis is linked to the homeostatic regulation of sleep. The importance of non-REM sleep in general was also demonstrated with the l-[1-^14^C]leucine method in a study of fetal sheep [307]. The radioligand was infused continuously for a six-hour period, and epochs of REM and non-REM sleep were identified after two hours of infusion. Protein synthesis in non-REM sleep was significantly greater than REM sleep.

### 5.5. Sleep, Protein Synthesis and Plasticity

The primary reason that it is important to also study protein synthesis during normal sleep is that this approach allows one to discuss the relationship between protein synthesis and sleep from a functional perspective. Substantial evidence exists for the role of protein synthesis in memory consolidation [310], and although direct evidence that supports a relationship between sleep, protein synthesis and memory consolidation is scarce, the temporal window where protein synthesis inhibitors can affect memory consolidation is similar to the temporal window where REM sleep deprivation can affect memory consolidation [136,311]. A few studies provide some indirect evidence.

Gutwein and colleagues [287] studied the relationship between protein synthesis and operant conditioning (*i.e.*, episodic declarative memory) using one-trial inhibitory avoidance training. Animals were placed into one compartment of an apparatus with two compartments. When the animal crossed into the second compartment, it received an electric shock. After a variable delay period, the animal was placed into the first compartment again, and the investigators measured the latency (maximum of 300 s) to cross into the second compartment. Anisomycin was administered immediately after encoding, and retrieval was tested after the following delay periods: 1 min, 15 min, 30 min, 45 min, 1 h, 3 h, 6 h, 9 h and 72 h. The presence of memory retention was indicated by latencies to cross into the second compartment that were not different from a saline control administration. Memory retention was decreased at the 1-min time point, an effect that was likely due to the drug’s effects on locomotion. For the subsequent delay periods, memory retention was essentially normal until three hours post-encoding, and decrements were observed for all remaining delay periods. This is the same time period during which REM deprivation affected performance. The authors speculate that decreases in protein synthesis during sleep immediately following memory encoding may have interfered with the sleep-dependent transfer of the memories from short-term to long-term storage. Within this paradigm, it would be interesting to test this idea further by measuring protein synthesis during sleep in each group and correlating the decrease in protein synthesis with the differences in long-term memory.

Smith and colleagues [312] performed a similar study using a two-way shuttle avoidance task. This task uses an apparatus with two compartments: A and B. Animals were placed in Compartment A. For each trial, a light was transiently illuminated in Compartment A, and the door that separated the compartments was opened. The animal was given 10 s to move to Compartment B (dark compartment), and if it did not, it received an electric shock. Performance at encoding (50 trials) and retrieval (20 trials) was indexed by the percentage of trials where the animal avoided the electric shock. The investigators administered anisomycin at different times in their groups, so that its effects were localized to a series of unique three-hour periods: 6–9 h post-encoding, 9–12 h post-encoding and 12–15 h post-encoding. Retrieval was tested at the end of each three-hour period. Although sleep was not measured in these animals, the investigators chose this manipulation, because prior results indicated that 9–12 h post-encoding on this task was the window in which REM sleep occurred and in which REM sleep deprivation impaired subsequent retrieval. They hypothesized that only animals that received anisomycin at 9–12 h post-encoding would show memory retrieval deficits. This hypothesis was supported; so, similar to the results from Gutwein and colleagues, these results indicate that protein synthesis during sleep supports memory consolidation during a critical REM window, but this window instead occurs 9–12 h after encoding. The differences in these studies may be due to the task that was used. The two-way shuttle avoidance task is a classical conditioning task (*i.e.*, a nondeclarative procedural memory); the electric shock is the unconditioned stimulus; the light is the conditioned stimulus, and moving to the dark compartment is both the unconditioned and conditioned response. In contrast, the one-trial inhibitory avoidance task used by Gutwein and colleagues is an episodic declarative memory task. In other words, the critical REM window for the consolidation of a declarative memory may take place from zero to 3 h after encoding, whereas the critical REM window for consolidation of a non-declarative memory may take place from nine to 12 h after encoding.

A more direct demonstration of the role of protein synthesis in memory consolidation during sleep comes from a study of fear conditioning in mice [313]. With a classical conditioning approach, inescapable foot shock was paired with a unique odor; following training, the odor was delivered to the animal during sleep. Subsequently, the conditioned response, freezing, was significantly enhanced. However, administration of anisomycin to the basal lateral amygdalae prior to exposure to the unique odor during sleep attenuated the fear memory. These results suggest that memories can be strengthened or weakened during sleep depending on the presence or absence of protein synthesis, respectively.

In many ways, we have focused on memory in this article, but memory is only one process that depends on brain plasticity. In experiments in immature cats and monkeys, it has been shown that if one eye is deprived of sensory input, the responsiveness of neurons in the primary visual cortex to stimulation delivered to that eye is decreased. This adaptive response is known as ocular dominance plasticity, and the associated sensitive period peaks approximately at Postnatal Day 32 in cats. In a series of experiments in cats, Frank and colleagues [314] tested whether this form of plasticity is sleep dependent. They compared four groups of cats: (1) 6 h of monocular deprivation; (2) 6 h of monocular deprivation followed by 6 h of sleep; (3) 6 h of monocular deprivation followed by 6 h awake in the dark; and (4) 12 h of monocular deprivation. Monocular deprivation was carried out in the light, and during this time, cats were not permitted to sleep. Following treatment, the investigators tested the electrophysiologic responsiveness of neurons in the primary visual cortex to stimuli delivered to the non-deprived eye. They found a significantly greater shift in the responsiveness of neurons to stimuli delivered to the non-deprived eye for the monocular deprivation + sleep group compared to monocular deprivation alone and monocular deprivation + sleep deprivation groups. This difference was comparable to the additional plasticity engendered by another period of actual monocular deprivation (12 h of monocular deprivation). In other words, the offline plasticity that occurred during sleep was comparable to the online plasticity that was caused by an additional period of normal waking sensory stimulation received by the non-deprived eye. Additional studies in cats addressed the role of protein synthesis in this sleep-dependent consolidation of cortical plasticity [315]. These studies demonstrated that inhibition of mammalian target of rapamycin complex 1 (mTORC1)-regulated protein synthesis by intracortical administration of rapamycin prevented consolidation during sleep, but had no effect on plasticity during wakefulness. In addition, relying on previous results indicating increased phosphorylated extracellular signal-regulated kinase (ERK) during post-monocular-deprivation sleep [183], the same investigators demonstrated that an inhibitor of ERK activation also prevented consolidation during sleep, but had no effect on plasticity during wakefulness [316]. These results indicate that mTORC1- and ERK-regulated protein synthesis play a significant role in sleep-dependent cortical plasticity.

## 6. The Potential Role of Disordered Sleep in the Pathophysiology of Neurodevelopmental Disorders

For many years, sleep was thought to be reflective rather than causative of a wide variety of psychiatric and neurological disorders. If one treated the “primary” disorder, the sleep disorder would spontaneously resolve. This sentiment is slowly being replaced with the idea that the disordered sleep is not merely a symptom of an unrelated disorder, but that it can play a role in the progression of the primary disorder. At a minimum, it is clear that disorders of sleep can and should be treated. Whether sleep disorders can trigger other disorders represents the forefront of sleep research, and each potential relationship must be carefully examined based on theoretical plausibility and experimental evidence. Numerous studies indicate that the severity of disordered sleep correlates with the severity of behavioral symptoms (communication and social behavior) in patients with autism (selected references [13,14,15]), TSC [16], fragile X syndrome [17] and hyperactivity in patients with ADHD [18]. Given the known presence of disordered sleep in neurodevelopmental disorders, the known alterations in cellular processes that accompany sleep deprivation and the importance of those same cellular processes in the pathophysiology of neurodevelopmental disorders, we will now specifically examine the role of disordered sleep in the manifestation of neurodevelopmental disorders.

### 6.1. Sleepiness/Inattention

Sleepiness is defined as the propensity to sleep. Although fatigue is a common synonym, it is important to note that cognitive fatigue is more specifically defined as a lack of motivation. Excessive daytime sleepiness is the primary consequence of sleep deprivation. This includes chronic, partial sleep deprivation or acute, total sleep deprivation. The inability to sustain vigilance is one of the most profound cognitive dysfunctions that is associated with sleepiness [317]. It may be the case that patients with neurodevelopmental disorders are chronically sleep deprived and that their cognitive symptoms are exacerbated by deficits in attention associated with sleepiness.

### 6.2. Accumulation of Sleep Regulatory Substances

According to the theory of local sleep, sleep is triggered in a top-down, use-dependent manner, and the accumulation of adenosine and other sleep regulatory substances is localized to the regions of the cortex that underwent the most activity during wakefulness [318]. The regional accumulation of sleep regulatory substances may serve as a tag that promotes plasticity in those regions during the subsequent period of sleep. Even if we assume that patients with neurodevelopmental disorders sleep well enough so that they do not exhibit excessive daytime sleepiness or grossly disordered sleep as measured by polysomnography, it is possible that their sleep may have subtle alterations where the cellular tags cannot induce the normal plasticity that accompanies sleep. This is an interesting idea given the known alterations in these patients in the same cellular processes that sleep regulates.

Ideas about local sleep are closely connected to protein synthesis through the synaptic tag hypothesis, which has been used to explain protein-synthesis-dependent LTP [319]. This hypothesis states that stimulation at a particular synapse triggers both the insertion of a tag at that synapse and an outgoing signal that, in turn, triggers protein synthesis in the cell body. Proteins are then sequestered by the tagged synapses. An increase in protein synthesis during sleep in the stimulated neurons may depend on sleep regulatory substances as the tag or as the outgoing signal. This scenario, of course, assumes that sleep follows shortly after training/stimulation. If, on the other hand, sleep deprivation occurs, the presence of the same sleep regulatory substances could be maladaptive and actually cause a decrease in protein synthesis in stimulated neurons.

### 6.3. Cellular Stress

As presented earlier (Section 4.2.3), sleep deprivation leads to the upregulation of the UPR and oxidative stress pathways. Given that disordered sleep often occurs in patients with autism and other neurodevelopmental disorders, could disordered sleep lead to the development of ER stress and oxidative stress in autism? While the answer to this question is not known, we will present evidence that both ER stress and oxidative stress may play a role in autism-related disorders, and these stress events can lead to changes in plasticity.

Some mutations associated with the risk for autism (like mutations in cell adhesion molecule-1 and neuroligin 3) have been shown to lead to upregulation of ER stress *in vitro* [320]. Additionally, ER stress has also been found in both *in vitro* and *in vivo* studies of mouse models of TSC [321,322]. Activation of ER stress causes calcium release and subsequent downstream cellular signaling. Calcium release can generate excitotoxicity and eventual cell death through apoptosis [234]. Even if the calcium release in the cell does not trigger apoptotic death, it may still have excitotoxic effects in the cell, altering cellular signaling and ultimately leading to aberrant plasticity.

Though controversial, there is evidence that sleep deprivation can increase oxidative stress. One important function of sleep may be the reduction of ROS, and melatonin is one important mediator of this reduction. The presence of excessive ROS may lead to negative effects on LTP, memory formation and cortical plasticity. In addition, the induction of oxidative stress (by treatment with the oxidative stress inducer, paraquat) results in fragmented sleep patterns [323], suggesting a downward spiral of disordered sleep, leading to ROS, leading to further disorders of sleep. Oxidative stress has been reported in the pathophysiology of autism [324,325,326], TSC [321,322], fragile X [327] and Rett syndrome [328].

### 6.4. Myelination

As discussed (Section 4.2.2), sleep deprivation leads to reduced myelination, and white matter abnormalities are hypothesized to be important in the etiology of almost all of the neurodevelopmental disorders that are also characterized as having disordered sleep (autism [329,330], TSC [331,332], fragile X syndrome [333,334], Rett syndrome [335], Prader–Willi syndrome [336], Angelman syndrome [337], Williams syndrome [338] and ADHD [339]). It is possible that disordered sleep and white matter abnormalities in these disorders may be linked.

### 6.5. Synaptic Regulation

Increased dendritic spine density is one of the hallmarks of fragile X syndrome [178,340,341], possibly affecting connectivity in the brain. Bushey and colleagues showed that *Drosophila* overexpressing *dFmr1* had decreased sleep and reduced axonal branching and differentiation. Sleep deprivation in these flies did not affect spine number or dendritic branching [178]. The authors suggested that, in this model, synaptic pruning occurred independently of sleep and, therefore, reduced the need for sleep. Specifically, the sleep-dependent pruning of dendritic spines may require proper expression of FMRP [178].

In this review, we also discussed that the neuroligin gene family, involved in synapse formation, is differentially regulated in conjunction with sleep. Altered expression of neuroligin genes may be an important mechanism of pathology in neurodevelopmental disorders. The neuroligin genes have been implicated in the pathology of autism [190,191,192]. Up- or down-regulation of neuroligins has negative consequences for synaptic plasticity. Patients with mutations in neuroligins or neurexins (interacting with neuroligin in synapse formation) or in these genomic regions exhibit both autistic behavior and disordered sleep [342,343,344,345]. Fragile X syndrome is also linked to altered neuroligin expression. Neuroligin mRNAs are targets of FMRP [346,347], and correction of neuroligin-1 expression in the fragile X mouse model reverses deficits in social interaction [347]. Though mutations in neuroligin can only explain a small subset of autism, it is hypothesized that synaptic dysfunction and altered excitatory/inhibitory connections are common features of the disorder [348,349,350]. We suggest that sleep disorders associated with autism may contribute to altered neuroligin expression and, consequently, to abnormalities in synaptic regulation.

### 6.6. mTORC1

One important regulator of cell proliferation and differentiation is the mTORC1 pathway [351,352]. Phosphorylation of downstream mTORC1 targets is increased during sleep, suggesting activated mTORC1 [315]. Short-term sleep deprivation in *Drosophila* and mice leads to decreased mTOR [247] and p-mTOR, which are restored after recovery sleep [237]. mTORC1 signaling abnormalities are hypothesized to play a causative role in several syndromic forms of autism [353]. Principally, mTORC1 is regulated through the protein products involved in TSC [354,355], leading to increased mTORC1 activity in patients [356]. Altered mTORC1 signaling during development can lead to altered brain connectivity and altered synaptic function [348]; both of which may be abnormal in autism [357]. It is possible that sleep abnormalities in autism may lead to altered mTORC1 signaling, which, in turn, may lead to altered connectivity and synapse formation.

### 6.7. Protein Synthesis

Many of the single gene disorders related to autism are caused by pathways involved in the regulation of protein synthesis and degradation [357]. In TSC, mTORC1 activity is increased. In fragile X syndrome, FMRP, a translational repressor, is absent. In Angelman syndrome, an E3 ubiquitin ligase involved in protein degradation is absent. In Rett syndrome, MECP2, which regulates the transcription of many genes, is altered. These genetic disorders suggest that rates of protein synthesis and degradation must be in a tight balance in order to maintain normal cell function. Dysregulated protein synthesis may lead to an improper balance of excitatory/inhibitory connections [357]. Additionally, overabundant protein synthesis can lead to the activation of the UPR, and too little protein synthesis can result in impaired myelination and cellular signaling.

Studies suggest that overall rates of protein synthesis in the brain are increased during sleep [307,308,309]. How does this relate to the hypothesis that patients with autism have increased protein synthesis, but disordered sleep? Increased protein synthesis during sleep has been primarily observed during SWS. REM sleep is often decreased in neurodevelopmental disorders [33,34,41,51,63,64,70,99,100,112,358], but SWS is reported to be increased in some cases [63,70,99,110,114]. Perhaps stage-specific changes in sleep and abnormalities in protein synthesis are linked with consequent effects on other cellular events, leading to abnormalities in plasticity and behavioral deficits. Third, EEG measures of sleep may lack the spatial resolution needed. Fourth, whether a contradiction exists at all depends on the temporal precedence assigned to each process. In other words, patients with neurodevelopmental disorders may sleep less, because they have a lower demand for sleep-dependent plasticity. This pathology may occur in a continuum across the sleep-wake cycle, where a lack of accumulation of normal plasticity markers during wakefulness causes a decreased demand for sleep and a decrease in protein synthesis or another cellular process during sleep. This is perhaps best illustrated by the short sleeping *dFmr1* hypermorph *Drosophila* model [65].

## 7. The Potential Role of Disordered Sleep in the Pathophysiology of Neurodegenerative Diseases

At the opposite end of the developmental spectrum, disorders of sleep are frequently noted in many age-related neurodegenerative diseases. It is possible that the hypotheses used in the context of neurodevelopmental disorders are equally applicable to neurodegenerative diseases. As the focus of this article is sleep and neurodevelopmental disorders, this section will not be a comprehensive review of sleep and neurodegenerative diseases.

Although disordered sleep is common in older populations, it is important to consider that disordered sleep in older populations is not necessarily normal; if closely examined, many sleep issues in the aged simply represent undiagnosed sleep disorders [359]. Nevertheless, it is observed that as age increases, sleep becomes increasingly shallow and irregular [360] and a higher percentage of time is spent in N1 sleep over SWS [361]. Major changes in N2 sleep and REM sleep occur only with advanced age [362,363], though small alterations in N2 sleep spindles and K-complexes do occur progressively over time (for a review, see [364]). Furthermore, impaired sleep may be further exacerbated by other co-morbid physical or mental conditions and medications taken, because nearly 50% of middle aged to older adults exhibit at least one symptom of insomnia [365].

Alzheimer’s disease (AD) is a progressive, age-related neurodegenerative disease that affects as many as five million adults in the United States and is the fifth leading cause of death in adults aged 65 and older [366]. As the most common form of dementia in adults, the disease primarily manifests as progressive memory impairment, dysfunction in temporal and spatial orientation, progressive apraxia and ataxia, behavioral changes and dementia. AD pathology includes amyloid and neuritic plaques, neurofibrillary tangles and neuronal degeneration. A primary genetic risk factor for AD is the presence of the ε4 allele in the apolipoprotein E (ApoE) gene [367], and its presence is directly associated with the incidence of cognitive impairment seen in elderly individuals with mild cognitive impairment (MCI) [368]. MCI, a form of early-onset dementia, has been generally accepted as a prodromal stage of AD (e.g., [369]).

Disordered sleep is present in most neurodegenerative diseases [360], and AD is no exception. AD patients show increased arousals during sleep, resulting in more N1 sleep and a reduction in the percentage of SWS. Additionally, increased REM sleep latency and decreased REM sleep time are characteristic of AD [370,371,372,373,374,375]. It is possible that the widespread degeneration of the cholinergic basal forebrain in AD underlies REM disturbances in light of the dependence of REM sleep on the cholinergic system [376]. Furthermore, in AD, the sleep spindles and K-complexes that are characteristic of N2 sleep are fewer in number, of lower amplitude, are poorly formed and are shorter in duration [377,378,379]. All non-REM stages become nearly indistinguishable from one another and from wakefulness, due to diffuse slow-wave activity that is characteristic in waking and sleeping in advanced AD [376]. Interestingly, as many of these characteristics suggest, sleep phenotypes in AD present as an accelerated form of aging [360]. Sleep disturbance, both objective and subjective, have also been reported in patients with MCI years before the development of full AD [380,381,382,383,384]. However, it is not yet known whether disordered sleep precedes the development of MCI.

Sleep impairment has long been implicated in the progression of the cognitive and behavioral sequelae of AD given reports that the extent of abnormal sleep is directly associated with the severity of dementia [372,378,385,386,387]. Therefore, we will briefly discuss two general mechanisms by which the cellular alterations that accompany sleep disturbances may lead to AD.

As previously described (see Section 4.2.3.1), one function of sleep could be to clear waste products and metabolites through a mechanism that involves the turnover of interstitial fluid [230]. Specifically, there is a higher clearance rate of radiolabelled amyloid-beta from the interstitial space in sleep compared to wakefulness. Recent evidence also suggests that the duration of wakefulness is positively associated with the growth of amyloid-beta deposits [388]. It has been suggested that the increased duration of wakefulness, due to impaired and fragmented sleep in MCI and AD, may be associated with the sub-cellular cascades involved in amyloid-beta deposition for years before producing symptoms of cognitive impairment [380]. This is consistent with the observation that amyloid-beta in cerebrospinal fluid correlates with sleep disturbances in the preclinical stage of AD [389].

Additionally, as previously described (see Section 4.2.3), chronic, partial sleep deprivation has deleterious effects on neuronal health, due to accumulating ER stress and oxidative stress. Sleep deprivation renders the ER ineffective at coping with cellular stress, which increases the frequency of protein folding errors, promotes protein aggregation, encourages ER-induced proapoptotic pathways and downregulates the UPR, thus cyclically contributing to more ER stress [390,391]. Aging limits the adaptive ability of the cell following sleep deprivation. The oxidative response following sleep deprivation was heightened in older animals [264]. Aged mice also have reduced basal expression of BiP and do not show elevated levels of BiP in response to sleep deprivation. In addition, sleep deprivation did not lead to increased levels of p-eIF2α, but does result in a significant increase in multiple proapoptotic markers of ER stress (CHOP and caspase-12) [390,392]. These results suggest that aged mice have an impaired adaptive UPR pathway and are more likely to initiate apoptosis associated with ER stress [393,394,395].

Disadvantageous ER signaling, due to ER stress and protein accumulation, are characteristic of numerous neurodegenerative diseases. Unsurprisingly, this is also true of AD in light of the amyloid and neuritic plaques and neurofibrillary tangles present in the AD pathology [391,396,397]. Since sleep impairments and ER stress are noted in MCI prior to the development of AD, prolonged disordered sleep may contribute to neurodegenerative disease progression.

Examination of the brains of AD patients also demonstrates significant oxidative damage associated with the accumulation of amyloid-beta plaques and neurofibrillary tangles [398]. Interestingly, the production of melatonin, an essential sleep regulator and antioxidant, is downregulated by as much as 80% in the elderly and further in populations with dementia [273,399].

Clearly, sleep is abnormal in patients with neurodegenerative diseases. Given the link between amyloid-beta deposits and wakefulness and the effects of sleep deprivation on the same sub-cellular processes that are altered in neurodevelopmental disorders, it is reasonable to suggest that disordered sleep is also important in the progression of neurodegenerative diseases.

## 8. Future Studies

Expanding this research area into humans is perhaps the most important new direction in which it could proceed. In fact, positron emission tomography (PET) studies of protein synthesis have been performed in humans and non-human primates for some time using l-[1-^11^C]leucine as the radioligand. This line of research began with the creation of a correction for unlabeled leucine from proteolysis of tissue proteins [400], the validation of the correction in rhesus monkeys [400] and the measurement of the sensitivity and stability of the technique in rhesus monkeys [401], and it culminated with the application of the technique to humans and the establishment of normal values, variability and reproducibility in humans [402]. If this technique were to be combined with a sleep-dependent memory study, choosing the most appropriate task would be critical. It should be a task in which the changes in the brain that occur during consolidation are restricted to a single region. For example, the Texture Discrimination Task is thought to depend on changes in the primary visual cortex, because performance gains based on training in one visual field do not transfer to another visual field [403]. This task is sleep-dependent, and protein synthesis during sleep has been proposed to underlie these sleep-dependent benefits [404]. The measurement of protein synthesis during sleep in the trained primary visual cortex hemisphere would not only test the theory of local sleep, but it would satisfy the five criteria of Frank and Bennington [405] for a truly integrative approach to the study of sleep-dependent plasticity: (1) the measurement of plasticity in the intact brain; (2) the study of an adaptive process, *i.e.*, visual perceptual learning; (3) the use of a well-understood cellular process, *i.e.*, protein synthesis; (4) the use of a rapidly learned task, *i.e.*, sleep-dependent texture discrimination learning has been demonstrated with daytime nap protocols [406,407]; and (5) the potential to illuminate the relationship between sleep and plasticity in other brain regions and at other developmental stages.

Although many have studied sleep in neurodevelopmental disorders, more research is warranted. For example, many studies were only conducted with retrospective questionnaires. It would seem prudent to include, at least, actigraphy and, at best, polysomnography. Sleep studies in the transgenic animal models of neurodevelopmental disorders would be particularly valuable, because once the presence of disordered sleep is established, one could test a variety of interventions in terms of their effectiveness at reversing not only sleep issues, but also other aspects of the phenotype [408], such as accompanying cognitive disabilities. Not only do sleep interventions represent an excellent treatment opportunity, they may be a necessary prerequisite before other interventions become effective. In other words, it may be the case that previous drug trials were unsuccessful because of the continued presence of the abnormalities of sleep. One unexplored pharmacological intervention to improve sleep is γ-hydroxybutyric acid, which is also known as sodium oxybate and Xyrem. This would be a relevant choice, because it has been suggested that its sedative effects may be mediated by increases in protein synthesis [409]. Indeed, administration of γ-hydroxybutyric acid triggers both SWS and growth hormone release [410]. This is consistent with the idea that protein synthesis is a critical component of sleep homeostasis. However, given that many of these neurodevelopmental disorders display GABAergic dysfunctions and given that the mechanism of action of γ-hydroxybutyric acid may involve GABA, it may be worthwhile to explore other novel hypnotics for use in neurodevelopmental disorders (e.g., hypocretin/orexin receptor antagonists). One choice for the timing of the intervention is in early development. Normalizing sleep early in development may mitigate the effects of the genetic mutation during the sensitive period when there are drastic sleep and synaptic changes.

Perhaps the biggest gap in this literature is the extreme paucity of sleep-dependent memory studies in patients with neurodevelopmental disorders. This research area should be expanded into other disorders and, most importantly, into other cognitive domains (consider social skills learning). In some neurodevelopmental disorders (e.g., autism), this is the feature of the disorder that sets it apart from other types of intellectual disabilities. There are no studies in any subject group that test whether social skills-learning is sleep-dependent. This cognitive domain is an ideal candidate to study within the context of sleep, because it is a complex cognitive skill that must be continually refined over a long period (*i.e.*, years). It is therefore likely that, over time, sleep serves to integrate an individual’s knowledge about social interactions into their existing schemas, which are mental representations about what we have come to expect about the world and which play a prominent role in modern theories of sleep-dependent memory [411].

## 9. Conclusion

Whether cellular stress from sleep deprivation leads to altered brain plasticity is controversial. Many of the studies on the cellular effects of sleep deprivation were done with acute, total sleep deprivation. In reality, the effects of chronic, partial sleep deprivation during a critical time in development and throughout a lifetime (as in the case with autism) are potentially different. Chronic, partial sleep deprivation may lead to accumulated changes, ultimately leading to, for example, neuronal cell death/dysfunction over time [271]. Because these are disorders of development, perhaps the timing of the abnormalities of sleep in the scope of critical brain maturation leads to more severe effects at different times. Additionally, there may be gene/environment interactions that alter the adaptive ability of the cell, where chronic, partial sleep deprivation in a normal individual may not cause substantial pathology, but chronic, partial sleep deprivation in a person predisposed to autism causes substantial neurobiological changes. Although this hypothesis may be less tenable in neurodevelopmental disorders with a Mendelian inheritance pattern, chronically-altered sleep may simply be more related to the severity of the cognitive symptoms in these cases.

Cell signaling and cortical connections are often altered in these disorders, and these alterations have unknown consequences on the sensitivity of the neurons to perturbations. Therefore, the combination of abnormalities of sleep and the altered brain conditions that are present in these neurodevelopmental disorders might lead to a synergistic and deleterious effect. These ideas are difficult to test, but doing so will likely yield significant advances in both basic and applied sleep research.
